# Supplementary material for: HIV testing and linkage to care—A case of a mobile diagnostic and counseling service in Mbeya, Tanzania; A quantitative study
Source: PLOS Glob Public Health. 2022 Aug 4;2(8):e0000448. doi: 10.1371/journal.pgph.0000448 (PMC10021411; doi:10.1371/journal.pgph.0000448)
Supplement: Attachment — Submitted filename: Response to Reviewers.docx [file pgph.0000448.s002.docx]

| **Editor’s comments** | **Authors’ response(s) to the Editor** |
| --- | --- |
| 1. Please update the completed 'Competing Interests' statement, including any COIs declared by your co-authors. If you have no competing interests to declare, please state "The authors have declared that no competing interests exist". Otherwise please declare all competing interests beginning with the statement "I have read the journal's policy and the authors of this manuscript have the following competing interests:" | “The authors have declared that no competing interests exist.” |
| 2. In the online submission form, you indicated that "The datasets used and/or analyzed during the current study are available from the corresponding author on reasonable request".    All PLOS journals now require all data underlying the findings described in their manuscript to be freely available to other researchers, either 1. In a public repository, 2. Within the manuscript itself, or 3. Uploaded as supplementary information.  This policy applies to all data except where public deposition would breach compliance with the protocol approved by your research ethics board. If your data cannot be made publicly available for ethical or legal reasons (e.g., public availability would compromise patient privacy), please explain your reasons by return email and your exemption request will be escalated to the editor for approval. Your exemption request will be handled independently and will not hold up the peer review process, but will need to be resolved should your manuscript be accepted for publication. One of the Editorial team will then be in touch if there are any issues. | “The datasets used and/or analyzed during the current study are uploaded as supplementary information.” |
| 3. Please amend your detailed Financial Disclosure statement. This is published with the article, therefore should be completed in full sentences and contain the exact wording you wish to be published.  ii). State the initials, alongside each funding source, of each author to receive each grant.  iii). State what role the funders took in the study. If the funders had no role in your study, please state: “The funders had no role in study design, data collection and analysis, decision to publish, or preparation of the manuscript.” | i) It is amended as per changes below.  ii) “AK was funded by the NIMR-MMRC through the institution seed grant project for young scientists.”  iii) “The funders had no role in study design, data collection and analysis, decision to publish, or preparation of the manuscript.” |
|  |  |
| **Reviewers’ Comments to the Author** | **Author’s response(s) to the Reviewers** |
| Reviewer #1: This is an interesting well written study from Tanzania. I enjoyed reading the paper. i have a few questions and suggestions. how do the authors reconcile "In Tanzania 52.2% of PLHIV 15 to 64 years, were aware of their HIV status and 90.9% 75 of those who knew their status were on HAART" mentionned in the introduction with their results should poor linkage to care. the discussion discusses the present results but if i understand clearly, the 90.9 % on care in Tanzania may reflect the majority of urban populations were care may be more accessible (i am speculating here). Perhaps this conundrum should be discussed is it a rural versus urban contrast? is it border areas? i think such a difference in the same country warrants more discussion.  The title of table 1 should specify the population described for the sake of table autonomy  table 2 on the right the column header anources total n (%) but there are only totals | Thank you for your comment.  “In Tanzania 52.2% of PLHIV 15 to 64 years, were aware of their HIV status and 90.9%”of those who knew their status were on HAART.” These findings are for the general population as per THIS 2016-2017.  Meanwhile “This study assessed the performance of a mobile mHTC in provision of HIV-testing and linkage to care of newly diagnosed PLHIV from KVPs”.  This study was conducted to understand the situation for KVPs due to their peculiarities compared to the general population.  Mbeya region is in the Southwestern highlands of Tanzania with an HIV prevalence of 9.3% ranking third as having the highest HIV prevalence in Tanzania; making it a priority region for intensified HIV/AIDS interventions in the country.  High population mobility associated with cross-border socio-economic activities, KVPs such as Female Sex Workers (FSWs), miners, long distance truck drivers, poverty and inadequate health facilities that can be accessed by KVPs are among the important factors driving the HIV epidemic in Mbeya.  We have specified the described population as “Key and Vulnerable Population”.  We have removed “(%)” and remained with only total (n). |
| Reviewer #2: This study assessed the performance of mobile HIV Testing and Counseling (mHTC) services in provision of HIV-testing and linkage to care of newly diagnosed PLHIV from Key and Vulnerable Populations (KVPs. This is a critical issue in Africa as linkage to ART service among PLHIV is of paramount importance to reach epidemic control. There is great need o establish why linkage to ART services is low and to also propose solutions to improve it. This article has thoroughly investigated the issue and identified some issues causing the low linkage. | Thank you very much for your comment. |
| Reviewer #3: This effort by the authors is a welcome contribution on the subject of linkage to HIV care from the perspective of enhancing uptake of HIV testing through new technologies and more user-friendly platforms in Tanzania and other countries with generalized HIV epidemics. The findings from the study were clear to me and I found them easy to follow. The abstract was well-written.  Major comments: a) Introduction:  There is insufficient literature review of like-minded studies to properly contextualize this study and establish an entry point for the authors. Please bolster your literature review credentials in an additional short paragraph.  b) The data analysis section is woefully under-specified. Please add more detail on the variables assessed (even at the risk of repetition). As it currently stands, the data analysis procedures and parameters are scanty indeed.  c) Discussion: A similar challenge here is insufficient juxtaposition of the authors' own findings with those from other authors say in Uganda, Nigeria, South Africa or Zimbabwe. Although they refer to some previous efforts by other authors, this is effort could be further strengthened to better link the authors findings to those of their peers in the same line of research.  Minor comments: a) Please reduce the use of brackets in your introduction and endevour to use free flowing text as much as possible. A case in point are lines 59-60.  b) Line 69: I would write.. Despite massive investments...  c) Methods: The study design is not explicitly stated. There are multiple sub-headings lumped together that begin this chapter which appears inappropriate. | “In Tanzania 52.2% of PLHIV 15 to 64 years, were aware of their HIV status and 90.9% of those who knew their status were on HAART (6); more innovative and client friendly modalities for HIV-testing and early initiation of therapy are needed in order to meet the United Nations Programme on HIV/AIDS (UNAIDS) 90-90-90 targets. Among the important factors driving the HIV epidemic in Mbeya are; high population mobility associated with cross-border socio-economic activities, Key and Vulnerable Populations (KVPs) such as Female Sex Workers (FSWs), miners, long distance truck drivers, poverty and inadequate health facilities that can be accessed by KVPs . The KVPs are a special group contributing to the burden of HIV in the region. The observed National AIDS Control Program (NACP) program performance data as per THIS 2016-2017 is likely to be different for KVPs. This study assessed the performance of mHTC services in provision of HIV-testing and linkage to care of newly diagnosed PLHIV from KVPs.”  “This study used retrospective data collected by the mHTC, some information such as factors affecting linkage were not captured i.e., stigma, discrimination, poverty, distance to the health facilities, while several other information like level of education had some missing data. The missing data is likely to have contributed to the findings observed in this study. Due to the nature of the mHTC services, some diagnosed PLHIV who were linked successfully after the mHTC have left a specific catchment area, their data could not be captured in this analysis. This study did not capture data on STIs, Tuberculosis and cervical cancer.”  We have also specified this in the limitation section part of the article.  In the period under investigation, the mHTC demonstrated an HIV-positivity of 3.17% in this rural setting. Our findings are closely comparable with the national data from the NACP published in 2017, indicating 4.3% prevalence for rural and 7.2% for urban areas (11). However, it is likely that there is significant variation in the regional data for the GP and KVPs in the rural areas of Tanzania. Among the important factors driving the HIV epidemic in Mbeya are high population mobility associated with cross-border socio-economic activities, KVPs such as FSWs, miners, long distance truck drivers, poverty and inadequate health facilities that can be accessed by KVPs. The KVPs are a special group contributing to the burden of HIV in the region. The observed NACP program performance data as per THIS 2016-2017 is likely to be different for KVPs. Our findings showed that HIV prevalence among KVPs in rural areas is lower as compared to the national prevalence in the same period, the observed difference could be explained by the fact that; although mHTC was targeting KVP clients but also GP who needed this service were not excluded upon request and therefore diluting the data on HIV prevalence; The limited data available shows close similarity with findings from another study in Mbeya, that indicated lower HIV infections in rural areas as compared to urban (12). Another study among randomly selected household members in rural areas in Kilimanjaro indicated that HIV prevalence in the targeted communities ranged from 1.4 -4.5% (13).  “Frequently cited barriers to linkage include; psychosocial like stigma and fear of disclosure; economic such as inability to afford transport costs, distance to health facilities and health system factors.”  “Despite massive investments”  “**Methods**  A retrospective review of the routine collected mHTC data was conducted between October-2016 to September-2018. The variables of interest in the analysis included age, sex, relationship status, level of education, HIV sero-status and linkage status. Linkage was defined as the confirmation of HIV-positive diagnosis and first HIV-specific clinic visit; as per WHO (8). Employing the “test and treat strategy”, newly diagnosed PLHIV were linked to Care and Treatment Center (CTC) of their choice by the mHTC team on the same day.  This study was conducted in rural areas of Mbeya region in the Southwestern highlands of Tanzania. The HIV prevalence for the whole region is 9.3% ranking third as having the highest HIV prevalence in Tanzania (6).The Region has 2,707,410 Population as per national census of 2012 (9). Unpublished data from the Mbeya Regional Medical Office’s, shows the health care infrastructure consists of 15 hospitals, 25 Health centers and 276 dispensaries; also 10 implementing partners working on HIV (10). The research work was a KVP focused study although General Populations (GPs) in need of the services were not denied for the reasons of the existing interactions between them. The targeted population was physically reached by mHTC and linkage to care of each newly diagnosed HIV positive client was done through physical escort to CTC of their choice. |
| Reviewer #4: Comment on PGPH-D-21-00487, Kway et al., This interesting paper by Kway et al explores the performance of mobile HIV testing and counseling services and linkage to care for newly diagnosed key and vulnerable populations. Linkage to care is an essential part of HIV-care targets and is a necessity for achieving the UNAIDS 90-90-90 targets. This paper focuses its analysis on key and vulnerable populations which are a known risk group for poor linkage and retention to care making this a relevant and needed analysis. There are comments listed below needed to be addressed prior to publication:  - The methods section needs to be revised to include more details about the study approach.    Information on the mHTC data needs to be clear.  What type of data/variables are available from mHTC?  How does it work in this population?    Where are patients referred for linkage using mHTC?    The population also needs to be clearly defined, a more specific definition of KVPs is needed.    The following sentence “High population mobility associated with cross-border socioeconomic activities, KVPs such as Female Sex Workers (FSWs), miners, long distance truck drivers, poverty and few health facilities near to target populations are 94 among the important factors driving the HIV epidemic in Mbeya” is more of a background statement than method.  More information on the catchment of Mbeya, how many clinics are serviced, how big the population is, who the KVP population is needed.  - Linkage needs to be clearly defined.  What time-period is this applicable?  A confirmation of HIV diagnosis to first HIV-clinic visit is very vague and leaves room for uncertainty on the time-frame in which linkage can occur.  In the era of test and treat a 2-week delay in linkage is substantial and it is unclear in the methods what period of time was allowed from first diagnosis to linkage.  - Data collection section needs major revision.  It is unclear if the 25,248 records were only from KVPs as exclusion only mentions those who were aware of their status prior to mHTC.  How did they determine if someone already knew their HIV status prior to mHTC?  Again, what does mHTC mean in this context?  Text message for appointment?  It needs to be absolutely clear to the reader what mHTC involves.  - Data analysis section is weak.  Sentence stating decision to categorize variables was based on previous studies but with no references cited in text.  More details are needed on the outcome and exposure variables.  Assuming linkage was binary (yes/no) based on their definition above, again it is not clear whether adjustment was done for calendar year, time, facility among other key variables.  The authors use log-binomial regression but it is unclear what the linear predictor in their model is. Why did they choose log-binomial?    - The authors do well to show table 1 and 2 with socio-demographic factors but it is unclear how they selected these. Are there other available variables?  The associations seen in the table may also be due to the number of various factors. Women more likely to be positive is ambiguous as it is unclear what proportion of women in the cohort were sex workers a known high risk group. A variable for type of key population (sex worker, truck driver, fisherman etc.) is necessary.  - The following sentence is unclear- Descriptive analyses revealed that of the diagnosed HIV-positive clients linked to-care 136 5/6 (83.33%) were children below 15 years, 238/450 (52.89%) were females, 39/64 (60.94%) 137 were widows.  Is there a reason why authors highlight widows? What is the rationale for marital status to be associated to linkage?    The inclusion of children (younger than 15) makes this analysis more complex to interpret. The association that those younger than 15 are more likely to link is an entirely different question as pediatric and adolescent HIV programs have their own clinics, practices, approaches when compared to adults. The small sample size 5/6 children linking could also be overestimating this association.  - The predictors to linkage of care (table 3) is not clear. Would rather state factors associated with linkage to care not predictors especially in this retrospective approach.  The lack of inclusion of other key variables makes this analysis weak. A strength would be including treatment variables and some longitudinal data since the analysis is based on data from an electronic medical database.    Linkage could be better defined and even care engagement and retention could be explored.  There are no strong conclusions from this analysis and this makes the overall message of the paper weak.    Lack of data on the clinics patient linked to care, how they linked, when they linked make the analysis very hard to interpret.  Major revisions are needed here.  Minor comments: - The introduction could be strengthened by incorporating a few sentences on KVPs and what linkage to care, including the 90-90-90 cascade looks like in this population and context.  Statistics are briefly mentioned on these targets in the general 15-64y population; however, more effort could be made to include context specific challenges to linkage to care.    A more detailed explanation of mobile health testing and counseling could also be introduced in the background. | mHTC data/variables includes age, sex, relationship status, level of education, HIV sero status, linkage status. The independent variables of interest in the analysis included age, sex, relationship status and level of education. HIV sero-status and linkage status were used as outcome variables.  “In the efforts to curb the HIV epidemic in the Southern Highlands regions of Tanzania, the National Institute for Medical Research-Mbeya Medical Research Centre (NIMR-MMRC) mobile laboratory has been implementing a “mobile HIV Testing and Counseling service (mHTC)” with funds from PEPFAR since 2009. Visiting between 8-12 Key and Vulnerable Populations (KVPs) sites every 3 months in Mbeya. mHTC was coupled with health education and screening for Sexually Transmitted Infection (STIs), Tuberculosis as well as cervical cancer.”  “The targeted population was physically reached by mHTC and linkage to care of each newly diagnosed HIV positive client was done through physical escort to CTC of their choice.”  “Employing the “test and treat strategy”, newly diagnosed PLHIV were linked to Care and Treatment Center (CTC) of their choice by the mHTC team on the same day.”  “Key populations are special groups characterized by specific higher-risk behaviors, posing them at increased risk of HIV regardless of the local context. Comprising of men who have sex with men, people who inject drugs, people in prisons and other closed settings, sex workers and transgender people (7). Vulnerable populations are groups of people who are more prone to HIV infection in certain contexts, such as adolescents girls, mobile workers etc (7).”  The text has been modified.  “This study was conducted in rural areas of Mbeya region in the Southwestern highlands of Tanzania. The HIV prevalence for the whole region is 9.3% ranking third as having the highest HIV prevalence in Tanzania (6).The Region has 2,707,410 Population as per national census of 2012 (9). Unpublished data from the Mbeya Regional Medical Office’s, shows the health care infrastructure consists of 15 hospitals, 25 Health centers and 276 dispensaries; also 10 implementing partners working on HIV (10).”  “Linkage was defined as the confirmation of HIV-positive diagnosis and first HIV-specific clinic visit; as per WHO (7). Employing the “test and treat strategy”, newly diagnosed PLHIV were linked to Care and Treatment Center (CTC) of their choice by the mHTC team”.  “Records of 25248 individuals from KVPs who received HIV test through mHTC services between October-2016 to September-2018 were extracted from the database”.  The research work was a KVP focused study although GPs in need of the services were not denied for the reasons of the existing interactions between them.  KVPs who had taken HIV test within one month were regarded as being aware of their status and did not receive HIV-test.  “In the efforts to curb the HIV epidemic in the Southern Highlands regions of Tanzania, the National Institute for Medical Research-Mbeya Medical Research Centre (NIMR-MMRC) mobile laboratory has been implementing a “mobile HIV Testing and Counseling service (mHTC)” with funds from PEPFAR since 2009. Visiting between 8-12 Key and Vulnerable Populations (KVPs) sites every 3 months in Mbeya. mHTC was coupled with health education and screening for Sexually Transmitted Infection (STIs), Tuberculosis as well as cervical cancer.”  “The targeted population was physically reached by mHTC and linkage to care of each newly diagnosed HIV positive client was done through physical escort to CTC of their choice.”  The text was modified.  The independent variables of interest in the analysis included age, sex, relationship status and level of education. HIV sero-status and linkage status were used as outcome variables.  Thank you for the comment, The analysis was limited to the variables in the dataset. Other variables which could have been helpful to analyze include facility type the client were enrolled, disclosure status was not collected. We have included the calendar year in the modified analysis.  “This study used retrospective data collected by the mHTC, some information such as factors affecting linkage were not captured i.e., stigma, discrimination, poverty, distance to the health facilities, while several other information like level of education had some missing data. The missing data is likely to have contributed to the findings observed in this study. Due to the nature of the mHTC services, some diagnosed PLHIV who were linked successfully after the mHTC have left a specific catchment area, their data could not be captured in this analysis. This study did not capture data on STIs, Tuberculosis and cervical cancer.”  We have also specified this in the limitation section part of the article.  Thank you for the comment, generalized linear model with binomial family and log link (log-binomial regression) was used because the prevalence of outcome was more than 10% but we have edited the resulting parameter to prevalence ratio instead of risk ration because the design was not a cohort.  It has been specified in the limitation section part of the article.  It has been specified in the limitation section part of the article.  Widows have been highlighted because in the relationship status they were leading in being successfully linked to care.  “This finding could possibly be explained by the fact that widows unlike the married women, do not need to request permission for linkage from their partners.”  Your point is very valid; the age group less than 15 years was not used as reference group in the analysis to avoid over estimation.  The text has been modified  “The approach of mHTC services started with provision of HIV-testing and ended with linkage to care of newly diagnosed PLHIV from KVPs.”  Among the limitation of this study is its retrospective nature. It was only possible to include relevant variables that were available within this context.  “Linkage was defined as the confirmation of HIV-positive diagnosis and first HIV-specific clinic visit; as per WHO. Employing the “test and treat strategy”, newly diagnosed PLHIV were Physically linked to Care and Treatment Center (CTC) of their choice by the mHTC team on the same day”.  “This study assessed the performance of mHTC services in provision of HIV-testing and linkage to care of newly diagnosed PLHIV from KVPs.”  “The approach of mHTC services started with provision of HIV-testing and ended with linkage to care of newly diagnosed PLHIV from KVPs.” It does not capture any patient data from the database at the health facilities.  “This study assessed the performance of mHTC services in provision of HIV-testing and linkage to care of newly diagnosed PLHIV from KVPs.”  “The approach of mHTC services started with provision of HIV-testing and ended with linkage to care of newly diagnosed PLHIV from KVPs.” “Employing the “test and treat strategy”, newly diagnosed PLHIV were linked to Care and Treatment Center (CTC) of their choice by the mHTC team on the same day.”  It does not capture any patient data from the database at the health facilities.  “Among the important factors driving the HIV epidemic in Mbeya are; high population mobility associated with cross-border socio-economic activities, KVPs such as Female Sex Workers (FSWs) and their clients, miners, long distance truck drivers, poverty and inadequate health facilities that can be accessed by KVPs. The KVPs are a special group contributing to the burden of HIV in the region. The observed National AIDS Control Program (NACP) program performance data as per THIS 2016-2017 is likely to be different for KVPs.”  “In the efforts to curb the HIV epidemic in the Southern Highlands regions of Tanzania, the National Institute for Medical Research-Mbeya Medical Research Centre (NIMR-MMRC) mobile laboratory has been implementing a “mobile HIV Testing and Counseling service (mHTC)” with funds from PEPFAR since 2009. Visiting between 8-12 Key and Vulnerable Populations (KVPs) sites every 3 months in Mbeya. mHTC was coupled with health education and screening for Sexually Transmitted Infection (STIs), Tuberculosis as well as cervical cancer.” |
| Reviewer #5:  Methods Mention the parameters of interest Define the older age group, young children, adults  Results The majority of the clients were in the age group 25-45 51.71% (put the percentage in brackets). It's easier to read.  Table 1: put the n. Complete the title: socio-demographic characteristics of the study population in general  Not categorized: young children (to be defined) and mention that it is the same group as not applicable.  Gender and sex used interchangeably.  Table 2: fourth column: Total n (%): there is no percentage.  Under table 2: give the explanation of non applicable.  Discussion Too many results included in this part although they have already mentioned in the Results section.  In the discussion on the frequencies of seropositivity in relation to divorced clients and widowers: this is explained by the fact that the majority were women. It is not shown in the results.  In the conclusion, present the highlights. No longer resume results and do not refer to THIS 2016-2017. Give the conclusions of your work. | The text has been modified  **“**The majority of the clients were in the age group 25-45 (51.71%).”  We have specified the described population as “Key and Vulnerable Population”.  The text has been modified  The text has been modified  We have removed “(%)” and remained with only total (n).  The text has been modified  The text has been modified  The text has been modified  The text has been modified |

Please use the space provided to explain your answers to the questions above. You may also include additional comments for the author, including concerns about dual publication, research ethics, or publication ethics. (Please upload your review as an attachment if it exceeds 20,000 characters)
